# Novel drug targets for *Mycobacterium tuberculosis*: 2-heterostyrylbenzimidazoles as inhibitors of cell wall protein synthesis

**DOI:** 10.1186/s13065-017-0295-z

**Published:** 2017-07-24

**Authors:** Mohana Rao Anguru, Ashok Kumar Taduri, Rama Devi Bhoomireddy, Malathi Jojula, Shravan Kumar Gunda

**Affiliations:** 1 Department of Chemistry, College of Engineering, Jawaharlal Nehru Technological University Hyderabad, Kukatpally, Hyderabad, 500 085 India; 2 Department of Microbiology, Sri Shivani College of Pharmacy, Warangal, 506002 India; 3 Bioinformatics Division, Osmania University, Hyderabad, 500007 India

**Keywords:** Benzimidazole, Green synthesis, *Mycobacterium tuberculosis*, Antibacterial activity, Molecular docking studies

## Abstract

**Background:**

Multi drug-resistant and mycobacterial infections are a major public health challenge, leading to high mortality and socioeconomic burdens through worldwide. Novel therapeutics are necessary to treat the drug resistant strains, since no new chemical entities are emerged in the last four decades for the treatment of TB.

**Findings:**

A series of novel 2-heterostyrylbenzimidazole derivatives were synthesised by cyclisation of (3,4-diaminophenyl)(phenyl)methanone, cinnamic acid using glycerol in high yield. The molecular structures of target compounds (**5a**–**5n**) were confirmed by ^1^H and ^13^C NMR spectroscopy and mass spectrometry. Newly synthesized compounds were screened for anti-tubercular activity and the MIC was determined against *Mycobacterium tuberculosis* H_37_Rv by broth microdilution method using Lowenstein Jensen medium (LJ). These compounds docked into the active site of “Crystal structure of pantothenate synthetase in complex with 2-(2-(benzofuran-2-ylsulfonylcarbamoyl)-5-methoxy-1H-indol-1-yl)acetic acid” (PDB code, 3IVX). Auto dock 4.2 software was used for docking studies.

**Results:**

**5d**, **5e**, **5f**, **5g**, **5i**, and **5l** show better activity and the most active inhibitor of tuberculosis **5f** showed a promising inhibition of *M. tuberculosis* with MIC value of 16 μg/mL. The molecules functionalized with electron-donating groups (Cl, O, S, etc.) on different aromatic aldehydes (**5a**–**5n**) were found to be more active in inhibiting *M. tuberculosis.*

**Conclusions:**

On the basis of docking studies, **5f** has shown good affinity for the enzyme. Comparison was made with the binding energies of the standard drugs amoxicillin (−34.28 kcal/mol) and ciprofloxacin (−28.20 kcal/mol). Among all the designed compounds, the compound **5f** shows highest binding energy with two amino acid interactions Lys160, Val187 (−9.80 kcal/mol).

## Background

Tuberculosis (TB) along with HIV ranks a leading cause of death worldwide. In 2015, tuberculosis killed 1.4 million people and 10.4 million people are estimated to have fallen ill with TB [1]. TB mortality has fallen since 1990; however, the rise of multidrug-resistant (MDR) and extremely drug-resistant (XDR) strains of *Mycobacterium tuberculosis* represents a serious health challenge. As per RNTC, drug-sensitive TB can be treated by 6 months of chemotherapy with the current four-drug frontline regimen. MDR-TB can be cured with at least 18–24 months of therapy using four to six drugs, including a fluoroquinolone and one injectable agent is required [2, 3]. XDR strains of *M. tuberculosis* additionally are resistant to fluoroquinolones and at least one second-line drug [1]. About 3% of new cases and 20% of treated tuberculosis patients are infected with MDR-TB; among these, about 9% are XDR cases [1]. Thus, tuberculosis becomes a significant threat to global health. So, novel therapeutics are necessary to treat both drug susceptible TB and progressively common drug resistant strains since, no new chemical entities are emerged in the past four decades for the treatment of TB [4, 5].

It is known that most of the currently existing tubercular medications are constituted by the group of nitrogen heterocyclic compounds such as isoniazid, pyrazinamide, etc. Further, most of them are derived from pyridine and pyrazines [6]. In an attempt to look for better bioactive heterocyclic compounds containing nitrogen hetero atom (since most of antituberculosis compounds are based on either pyridine or pyrazines), our consideration curved in the direction of benzimidazole derivatives, as these compounds exhibit a wide spectrum of biological activities including antituberculous activity [7]. Specifically, this nucleus is a constituent of vitamin-B12 and many currently existing medications [8]. Almost all benzimidazoles with different heterocyclic substituents led to essential modification in their physico-chemical, metabolic and pharmacokinetic properties [9].

**Fig. 1:**
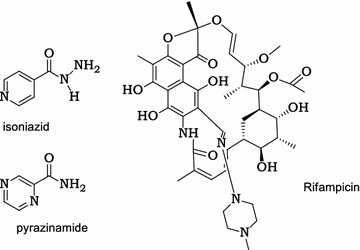
Currently available anti-tubercular drugs containing amide linkage

Only few reports are available in the literature on antituberculous activity of benzimidazoles [10, 11]. Further, literature survey revealed that most of the first-line anti-tuberculous drugs were constituted by amide linker (shown in Fig. 1). However, remarkable antitumor/anti proliferative/anticancer [12–15] activity of 1-substituted benzimidazole derivatives prompted us to carry out the cytotoxic activity. Taking into account of the functional group similarity of the amide linkage [16, 17] the structural similarity of the pyridopyrazine moiety, albendazole and thiabendazole [18, 19] are evaluated. With the aim of obtaining pharmacologically active compounds, we have envisioned that the benzimidazole scaffold could be a good starting material for the development of good MTB inhibitors. After extensive literature search, it was observed that, till date enough effort has not been made to combine these moieties as a single molecular scaffold and identify new candidates that may be of value, in designing new, potent and selective antitubercular agents.

Molecular docking studies were performed on binding site of pantothenate synthetase protein to study the binding mode of compounds. The results of both invitro and insilico studies clearly indicated that 2-heterostyrylbenzimidazole may serve as new drug candidates in the combat against *M. tuberculosis* protein (3IVX). In continuation to our efforts in this drug design paradigm, a library of (*E*)-phenyl-(2-styryl-1H-benzo[d]imidazol-6-yl)methanone derivatives have been synthesized and evaluated for their biological activity. Molecular docking studies using this protein target have also not yet been reported. Hence, molecular docking studies of the synthesized 1H-benzimidazoles were performed on protein PDB code 3IVX by means of molecular operating environment (MOE) software (Autodock 4.2). In the combat against multi-drug resistance, such insilico studies have played a key role in the identification of new drug targets and the designing of new scaffolds as novel drug candidates.

**Scheme 1:**
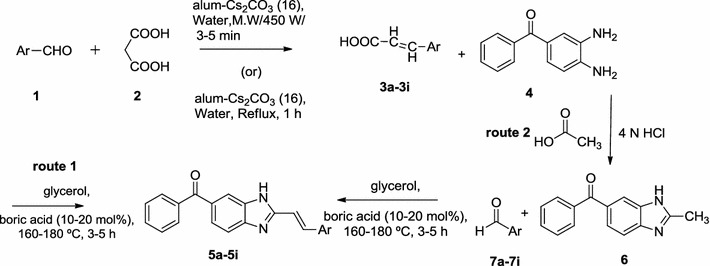
Synthesis of target compounds (**5a**–**5i**)

**Scheme 2:**
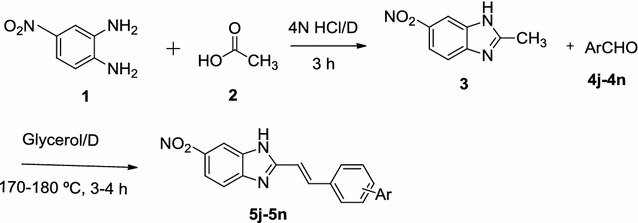
Synthesis of target compounds (**5j**–**5n**)

In view of this data, we reported the synthesis of 2-styrylbenzimidazole derivatives from O-PDA and cinnamic acids, 4-nitro benzene diamine, acetic acid (Schemes 1, 2) which possessed wide variety of biological activity encouraging antitubercular activity against *M. tuberculosis* H_37_Rv and MDR. In continuation of our efforts in this drug design paradigm, a library of (*E*)-phenyl-(2-styryl-1H-benzo[d]imidazol-6-yl)methanone derivatives have been synthesized and evaluated for molecular docking studies.

## Methods

A simple conventional method is followed to prepare all the compounds.

### Experimental

All the reagents used in the present study were obtained from commercial suppliers. All the solvents were freshly distilled before being used. Melting points were determined using a Buchi melting point B-545 apparatus and are uncorrected. TLC analyses were done on glass plates coated with silica gel GF-254 and spotting was done using iodine/UV lamp. IR spectra were recorded on a Perkin-Elmer model 446 instrument in KBr phase. H^1^ NMR were recorded by 400 MHz spectrometer, LC–MS spectrometer, model HP5989A. ^13^C NMR was recorded in DMSO using 100 MHz spectrometer.

### General procedure for the synthesis of 2-styrylbenzimidazole derivatives from O-PDA and cinnamic acids (route 1, Scheme 1)

An intimate mixture of *o*-phenylenediamine 1 (1.08 g, 10 mM) was dissolved in 10 mL of glycerol in a 250 mL round bottom flask. Triacetylborate (0.2 g, 10 mol) and cinnamic acids **3a**–**3i** (10 mM) are added and allowed the solution to boil at 160–180 °C for 3 h using dean-stark apparatus placed in oil-bath. The completion of the reaction was monitored by checking TLC. At the end of this period, the reaction mixtures was poured into ice cold water and adjust the pH of the solution to 8.0–10.0. Filter the compound and recrystallized by using suitable solvent.

### General procedure for the synthesis of 2-styrylbenzimidazole derivatives from 2-methylbenzimidazoles and aromatic aldehydes (route 2, Scheme 1)

An intimate mixture of 2-methylbenzimidazoles 6 (1.32 g, 10 Mm) was dissolved in 10 mL of glycerol in a 250 mL round bottom flask was added triacetyleborate (0.2 g, 10 mol) and corresponding aromatic aldehydes **7a**–**7i** (10 Mm) and allowed to boil at 160–180 °C for 3 h using dean-stalk apparatus placed in oil-bath. The completion of the reaction was monitored by checking TLC.

### General procedure for the synthesis of 2-styrylbenzimidazole derivatives from 4-nitro benzene di amine and acetic acid (Scheme 2)

4-Nitro benzene di-amine on reaction with acetic acid in 4 N HCl under Phillip’s conditions for 3 h gave the well-known 2-methylbenzimidazole [3]. 2-methylbenzimidazole [3] on condensation with benzaldehydes (**4j**–**4n**) at 170–180 °C using dean-stark apparatus placed in oil-bath. The completion of the reaction was monitored by checking TLC.

#### (*E*)-phenyl-(2-styryl-1H-benzo[d]imidazol-6-yl)methanone (**5a**)

Light orange crystals, yield (2.9 g, 88%), m.p 202–204 °C, IR (KBr, *ʋ*_max_ cm^−1^): 3401 (–NH), 2956 (=C–H), 1891 (C=N), 1610 (C=C), 1644 (C=O), ^1^H NMR (DMSO-*d*_*6*_/TMS, 400 MHz) δ_ppm_ 7.09–7.13 (d, 1H, –C=CH– vinylic proton, *J*_H-H_ = 16.4 Hz), 7.33–7.37 (d, 1H, –CH=C, vinylic proton, *J*_H-H_ = 16.4 Hz), 7.48–7.67 (m, 8H-aryl, 5H-phenyl), 10.2 (s, 1H, –NH of benzimidazole). ^13^C NMR (DMSO-*d*_*6*_, 100 MHz) δ_ppm_ 102, 106, 108, 110, 112, 120, 121, 122, 128, 129, 130, 132, 142, 148, 152, 190, MS (*m/z*): 325.2 (M^+^).

#### (*E*)-(2-(4-fluorostyryl)-1H-benzo[d]imidazol-6-yl)(phenyl)methanone (**5b**)

Black crystals, yield (3.0 g, 90%), m.p 178–180 °C, IR (KBr, *ʋ*_max_ cm^−1^): 3432 (–NH), 3178 (=C–H), 1909 (C=N), 1628 (C=C), 1700 (C=O). ^1^H NMR (DMSO-*d*_*6*_/TMS, 400 MHz) δ_ppm_ 7.09–7.13 (d, 1H, –C=CH– vinylic proton, *J*_H-H_ = 16.4 Hz), 7.3–7.9 (m, 8H-aryl, 4H-phenyl), 8.09–8.13 (d, 1H, –CH=C–, vinylic proton, *J*_H-H_ = 16.4 Hz), 10.2 (s, 1H, –NH of benzimidazole). ^13^C NMR (DMSO-*d*_*6*_, 100 MHz) δ_ppm_ 102.29, 106.24, 108.53, 110, 112, 120, 121, 122, 128.51, 129, 130, 132, 142, 148, 152, 190.91, MS (*m/z*): 343.2 (M^+^).

#### (*E*)-(2-(4-chlorostyryl)-1H-benzo[d]imidazol-6-yl)(phenyl)methanone (**5c**)

Brown crystals, yield (3.2 g, 90%), m.p 130–132 °C, IR (KBr, *ʋ*_max_ cm^−1^): 3426 (–NH), 2898 (=C–H), 1909 (C=N), 1601 (C=C), 1680 (C=O), ^1^H NMR (DMSO-*d*_*6*_/TMS, 400 MHz) δ_ppm_ 7.09–7.13 (d, 1H, –C=CH– vinylic proton, *J*_H-H_ = 16.4 Hz), 7.3–7.9 (m, 8H-aryl, 4H-phenyl), 8.09–8.13 (d, 1H, –CH=C–, vinylic proton, *J*_H-H_ = 16.4 Hz), 10.2 (s, 1H, –NH of benzimidazole). ^13^C NMR (DMSO-*d*_*6*_, 100 MHz) δ_ppm_ 102, 106, 108, 110, 112, 120, 121, 122, 128, 129, 130, 132, 142, 148, 152, 190, MS (*m/z*): 359.1 (M^+^).

#### (*E*)-(2-(2-(furan-2-yl)vinyl)-1H-benzo[d]imidazol-6-yl)(phenyl)methanone (**5d**)

Black crystals, yield (2.5 g, 82%), m.p 100–102 °C, IR (KBr, *ʋ*_max_ in cm^−1^): 3423 (–NH), 2921 (=C–H), 1642 (C=N), 1597 (C=C), 1617 (C=O), ^1^H NMR (DMSO-*d*_*6*_/TMS, 400 MHz) δ_ppm_ 6.6–8.1 (m, 8H-aryl, 3H-furanyl, 2H-vinylic protons, *J*_H-H_ = 16.4 Hz), 10.2 (s, 1H, –NH of benzimidazole). ^13^C NMR (DMSO-*d*_*6*_, 100 MHz) δ ppm: 111.00, 112.01, 118.01, 125.12, 128.45, 128.68, 128.91, 129.47, 130.43, 131.85, 132.27, 137.75, 139.96, 152.32, 195.27, MS (*m/z*): 370.2 (M^+^).

#### (*E*)-phenyl(2-(2-(thiophen-2-yl)vinyl)-1H-benzo[d]imidazole-6-yl)methanone (**5e**)

Light green crystals, yield (2.6 g, 80%), m.p 108–110 °C, IR (KBr, *ʋ*_max_ in cm^−1^): 3427 (–NH), 3060 (=C–H), 1633 (C=N), 1597 (C=C), 1614 (C=O). ^1^H NMR (DMSO-*d*_*6*_/TMS, 400 MHz) δ_ppm_ 6.6–8.1 (m, 8H-aryl, 3H-thiophenyl, 2H-vinylic protons, *J*_H-H_ = 16.4 Hz), 10.2 (s, 1H, –NH of benzimidazole). ^13^C NMR (DMSO-*d*_*6*_, 100 MHz) δppm 111.00, 112.01, 118.01, 125.12, 128.45, 128.68, 128.91, 129.47, 130.43, 131.85, 132.27, 137.75, 139.96, 152.32, 195.27, MS (*m/z*): 370.2 (M^+^).

#### (*E*)-(2-(2-(benzo[d][1,3]dioxol-5-yl)vinyl)-1H-benzo[d]imidazol-6-yl)(phenyl)methanone (**5f**)

Dark brown crystals, yield (3.2 g, 88%), m.p −240 °C, IR (KBr, *ʋ*_max_ in cm^−1^): 3422 (–NH), 2917 (=C–H), 1644 (C=N), 1575 (C=C), 1609 (C=O). ^1^H NMR (DMSO-*d*_*6*_/TMS, 400 MHz) δ_ppm_ 2.5 (s, 2H, –CH_2_), 6.2–6.26 (d, 1H, –C=CH–, vinylic proton, *J*_H-H_ = 16.4 Hz), 7.0–8.0 (m, 8H-aryl, 4H-phenyl, 1H-vinylic protons, *J*_H-H_ = 16.4 Hz), 9.8 (s, 1H, –NH of benzimidazole). ^13^C NMR (DMSO-*d*_*6*_, 100 MHz) δ_ppm_ 114.30, 115.04, 116.96, 125.42, 128.45, 128.68, 129.12, 129.30, 129.48, 129.59, 131.08, 132.22, 132.33, 133.76, 134.37, 137.58, 137.64, 137.74, 195.17, MS (*m/z*): 370.2 (M^+^).

#### (*E*)-(2-(2-(1H-indol-2-yl)vinyl)-1H-benzo[d]imidazol-6-yl)(phenyl)methanone (**5g**)

Light green crystals, yield (1.97 g, 92%), m.p 198–200 °C, IR (KBr, *ʋ*_max_ in cm^−1^): 3405 (–NH), 3101 (=C–H), 1894 (C=N), 1633 (C=C). ^1^H NMR (DMSO-*d*_*6*_/TMS, 400 MHz) δ_ppm_ 6.91–6.95 (d, 1H, –C=CH vinylic, *J*_H-H_ = 16.4 Hz), 7.16–7.18 (t, 1H, thiophenyl), 7.23–7.28 (q, 2H, phenylic), 7.44–7.45 (d, 1H, thiophenyl), 7.57–7.61 (q, 2H, phenylic), 7.67–7.68(d, 1H, thiophenyl), 7.92–7.96 (d, 1H, –CH=C–(vinylic), *J*_H-H_ = 16.4 Hz), 10.0 (s, 1H, –NH of benzimidazole). ^13^C NMR (DMSO-*d*_*6*_, 100 MHz) δ_ppm_ 114.47, 114.50, 122.87, 127.96, 128.51, 129.15, 129.48, 137.39, 140.33, 149.87, MS (*m/z*): 227.07 (M^+^).

#### (*E*)-(2-(4-nitrostyryl)-1H-benzo[d]imidazol-6-yl)(phenyl)methanone (**5h**)

Light yellow crystals, yield (3.2 g, 86%), m.p 210–212 °C, IR (KBr, *ʋ*_max_ in cm^−1^): 3410 –NH), 2960 (=C–H), 1680 (C=N), 1620 (C=C), 1670 (C=O). ^1^H NMR (DMSO-*d*_*6*_/TMS, 400 MHz) δ_ppm_ 7.0–8.3 (m, 8H-aryl, 4H-phenyl, 2H-vinylic protons, *J*_H-H_ = 16.4 Hz), 10.2 (s, 1H, –NH of benzimidazole). ^13^C NMR (DMSO-*d*_*6*_, 100 MHz) δ_ppm_ 102, 106, 108, 110, 112, 120, 121, 122, 128, 129, 130, 132, 142, 148, 152, 190, MS (*m/z*): 370.2 (M^+^).

#### (*E*)-(2-(2-nitrostyryl)-1H-benzo[d]imidazol-6-yl)(phenyl)methanone (**5i**)

Light yellow crystals, yield (3.2 g, 86%), m.p 210–212 °C, IR (KBr, *ʋ*_max_ in cm^−1^): 3410 (–NH), 2960 (=C–H), 1680 (C=N), 1620 (C=C), 1670 (C=O). ^1^H NMR (DMSO-*d*_*6*_/TMS, 400 MHz) δ_ppm_ 7.0–8.3 (m, 8H-aryl, 4H-phenyl, 2H-vinylic protons, *J*_H-H_ = 16.4 Hz), 10.2 (s, 1H, –NH of benzimidazole). ^13^C NMR (DMSO-*d*_*6*_, 100 MHz) δ_ppm_ 102, 106, 108, 110, 112, 120, 121, 122, 128, 129, 130, 132, 142, 148, 152, 190, MS (*m/z*): 370.2 (M^+^).

#### (*E*)-2-(4-fluorostyryl)-6-nitro-1H-benzo[d]imidazole (**5j**)

Brown crystals, yield (2.49 g, 88%), m.p 203–206 °C, IR (KBr, *ʋ*_max_ in cm^−1^): 3405 (–NH), 3101 (=C–H), 1894 (C=N), 1633 (C=C), 1340–1520 (NO_2_), ^1^H NMR (DMSO-*d*_*6*_/TMS, 400 MHz) δ_ppm_ 6.9–7.9 (q, 4H-aryl protons), 7.71–7.75 (d, 1H-vinylic proton, *J*_H-H_ = 16.4 Hz), 7.76–7.79 (q, 2H-aryl protons), 8.80–8.84 (d, 1H-vinylic proton, *J*_H-H_ = 16.4 Hz), 8.42 (s, 1H, ArH), 10.0 (s, 1H, NH, benzimidazole). ^13^C NMR (DMSO-*d*_*6*_, 400 MHz) δ_ppm_ 115.85, 115.93, 115.95, 116.06, 118.10, 129.57, 129.65, 131.73, 131.76, 132.09, 136.36, 142.63, 155.14, 161.47, 190.20, MS (*m/z*): 284.07 (M^+^).

#### (*E*)-2-(2-(1H-indol-2-yl)vinyl)-1H-benzo[d]imidazole (**5k**)

Light green crystals, yield (1.97 g, 92%), m.p 198–200 °C, IR (KBr, *ʋ*_max_ in cm^−1^): 3405 (–NH), 3101 (=C–H), 1894 (C=N), 1633 (C=C). ^1^H NMR (DMSO-*d*_*6*_/TMS, 400 MHz) δ_ppm_ 6.91–6.95 (d, 1H,=CH-vinylic proton, *J*_H-H_ = 16.4 Hz), 7.16–7.18 (t, 1H, thiophenyl proton), 7.23–7.28 (q, 2H, phenylic protons), 7.44–7.45 (d, 1H, thiophenyl proton), 7.57–7.61 (q, 2H, phenylic protons), 7.67–7.68(d, 1H, thiophenyl proton), 10.0 (s, 1H, –NH, benzimidazole). ^13^C NMR (DMSO-*d*_*6*_, 100 MHz) δ_ppm_ 114.47, 114.50, 122.87, 127.96, 128.51, 129.15, 129.48, 137.39, 140.33, 149.87. MS (*m/z*): 227.07 (M^+^).

#### (*E*)-6-nitro-2-styryl-1H-benzo[d]imidazole (**5l**)

Brown crystals, yield (2.49 g, 88%), m.p 203–206 °C, IR (KBr, *ʋ*_max_ in cm^−1^): 3405 (–NH), 3101 (=C–H), 1894 (C=N), 1633 (C=C), 1340–1520 (NO_2_). ^1^H NMR (DMSO-*d*_*6*_/TMS, 400 MHz) δ_ppm_ 6.9–7.9 (q, 4H-aryl protons), 7.71–7.75 (d, 1H-vinylic proton, *J*_H-H_ = 16.4 Hz), 7.76–7.79 (q, 2H-aryl protons), 8.80–8.84 (d, 1H-vinylic proton, *J*_H-H_ = 16.4 Hz), 8.42 (s, 1H- aromatic proton), 10.0 (s, 1H, –NH benzimidazole). ^13^C NMR (DMSO-*d*_*6*_, 400 MHz) δ_ppm_ 115.85, 115.93, 115.95, 116.06, 118.10, 129.57.

#### (*E*)-2-(2-(furan-2-yl)vinyl)-6-nitro-1H-benzo[d]imidazole (**5m**)

Yield 2.04 gm (80%); m.p 240 °C, IR (KBr, *ʋ*_max_ in cm^−1^): 3405 (–NH), 3101 (=C–H), 1894 (C=N), 1633 (C=C), 1340–1520 (NO_2,_). ^1^H NMR (DMSO-*d*_*6*_/TMS, 400 MHz) δ_ppm_ 6.5–7.8 (m, 3H-furanyl protons), 6.99–7.03 (d, 1H-vinylic proton, *J*_H-H_ = 16.4 Hz), 7.03–7.07 (d, 1H-vinylic proton, *J*_H-H_ = 16.4 Hz), 7.6–8.1 (m, 3H-aryl protons), 10.0 (s, 1H, –NH, benzimidazole). MS (*m/z*): 256.07 (M^+^).

#### (*E*)-2-(2-(benzo[d][1,3]dioxol-5-yl)vinyl)-6-nitro-1H-benzo[d]imidazole (**5n**)

Dark brown crystals, yield (3.2 g, 88%), m.p >240 °C, IR (KBr, *ʋ*_max_ in cm^−1^): 3422 (–NH), 2917 (=C–H), 1644 (C=N), 1575 (C=C), 1609 (C=O), ^1^H NMR (DMSO-*d*_*6*_/TMS, 400 MHz) δ_ppm_ 2.5 (s, 2H, –CH_2_), 6.2–6.26 (d, 1H, –C=CH–, vinylic proton, *J*_H-H_ = 16.4 Hz), 7.0–8.0 (m, 8 aryl, 4H-phenyl, 1H-vinylic protons, *J*_H-H_ = 16.4 Hz), 9.8 (s, 1H, –NH of benzimidazole). ^13^C NMR (DMSO-*d*_*6*_, 100 MHz) δ_ppm_ 114.30, 115.04, 116.96, 125.42, 128.45, 128.68, 129.12, 129.30, 129.48, 129.59, 131.08, 132.22, 132.33, 133.76, 134.37, 137.58, 137.64, 137.74, 195.17, MS (*m/z*): 370.2 (M^+^).

## *M. Tuberculosis*

### Materials and methods

#### Anti-mycobacterial agents

Test drugs included pyrazinamide (Z), rifampicin (R), streptomycin (S), ethambutol (E), and isoniazid (H) obtained by Sigma. According to CDC (1985) recommendations stock solution was prepared. The following concentrations were used for the screening antimicrobial agents: H: 0.05–3.2; R: 0.25–16; E: 0.5–32; S: 0.5–32 and Z: 12.5–800 mg/mL. All drugs were kept as a stock suspension of 1% in distilled water except for R that were dissolved in methanol, and stored at −25 °C.

#### LJ media preparation

Lowenstein-Jensen medium is used with fresh egg and glycerol for the isolation and differentiation of *M. tuberculosis*.

#### Preparation of Lowenstein-Jensen medium

Lowenstein-Jensen (LJ) medium is most widely used for tuberculosis culture. LJ medium containing glycerol favours the growth of *M. tuberculosis*.

## Middle Brook media preparation

### Preparation of liquid medium, 7H9 liquid medium preparation

Middlebrook 7H9 Broth is a liquid growth medium specially used for culture of Mycobacterium, notably *M. tuberculosis*. 7H9 supports the growth of mycobacterial species which is supplemented with nutrients such as glycerol, oleic acid, albumin and dextrose. Media is used for the preparation of inocula for anti mycobacterial assays. 7H9 broth supports the growth of mycobacterial species when supplemented with nutrients such as glycerol, oleic acid, albumin and dextrose, except for *M. tuberculosis* which is inhibited by glycerol. Cultures should be read within 5–7 days after inoculation and once a week thereafter for up to 8 weeks. Middlebrook broth is commonly used in the preparation of inocula for antimicrobial assays, biochemical tests (arylsufatase and tellurite reduction) and for maintenance of stock strains.

### Drug concentrations

#### Preparation of stock solution

50 mg of isoniazid was prepared by adding 5 mL sterile distilled water.

#### Working solution

0.5 mL from the stock solution is taken and 24.5 mL of distilled water is added and 0.1 mL of the drug is added to the media (conc. 0.2 µg/mL).

### New drug dilution

Compounds (1 mg) are soluble in 1 mL of the DMSO (con. 1 mg/mL), after dilutions final concentrations are 1000 µg/mL/1 mg/1 mL, 500 µg/mL, 250 µg/mL, 125 µg/mL, 62 mg/mL, 31 µg/mL, 15 µg/mL, 8 µg/mL, 4 µg/mL. Further, the dilution procedure in Step-1 label the test tubes as 1, 2, 3, 4, 5, 6, 7, 8, 9, 10. For MTB H_37_Rv (set A) and MDR strain (set B) separately, in Step-2 take 2 mL of stock solution in test tube no. 1 (in each set), concentration 1000 µg/ml, in Step-3, transfer 1 mL of the solution into test tube no. 2 (in each set), and dilute it with 1 mL of DMF (in each set). Now the concentration becomes one half to the first test tube (no. 1), i.e., 500 µg/mL. In Step-4, mix well the contents in test tube, and transfer 1 mL of the solution from test tube no. 2 into test tube no. 3 add 1 mL of DMF to the test tube no. 3 as mentioned in Step-3. Then we get the concentration of 250 µg/mL (test tube no. 3). In Step-5, repeat the dilution as mentioned in Step-3 and 4 up to test tube no. 9, and discard the 1 mL of solution from test tube no. 9. In Step-6, the test tube no. 10 remains control (only DMSO).

### Bacterial strains and cultures

All the clinical samples were collected from District Tuberculosis Center, MGM hospital, Warangal and inoculated on to the LJ solid medium and incubated for 8 weeks at Department of Microbiology, Sri Shivani College of Pharmacy, culture were identified as *M. tuberculosis* based on morphological and biochemical methods. 50 clinical isolates were preserved. Mono resistant strain of *M. tuberculosis* was identified by testing drug susceptibility for isoniazid (H) and it was taken as test strain and *M. tuberculosis H37RV* taken as a control strain for testing antimycobacterial activity of compounds. The bacterial strains were stored in Trypticase soy broth containing glycerol at −70 °C. All the strains were recovered on Lowenstein-Jensen medium for 21–28 days at 37 °C.

#### Preparation of inoculums

The isolates grown on Lowenstein Jensen medium (LJ) were sub-cultured in Middle Brook 7H9 broth [20–22] supplemented with OADC at 37 °C for 14–21 days. The bacterial suspension was homogenized by vortex shakeup and the turbidity was adjusted in agreement with tube which is the scales of McFarland no. 1 (3.2 × 10^6^ cfu/mL). The inoculum was prepared diluting the bacterial suspension in the proportion of 1:20 in Middle Brook 7H9 broth medium. This diluted suspension (100 μL) was used to inoculate. The mycobacteria were grown in Middlebrook 7H9 medium (HiMedia, India) supplemented with 10% ADC (HiMedia, India). Log phase cultures were centrifuged, washed twice with sterile saline and adjusted to McFarland standard corresponding to 1 × 10^7^ cfu/mL. The size of inoculum was confirmed by plating serial dilutions on Middlebrook 7H11 media (HiMedia, India) plates supplemented with 10% OADC (HiMedia, India). The plates were incubated for 4 weeks prior to CFU enumeration.

### Broth dilution method [23–25]

The MIC for *M. tuberculosis* H37Rv and drug resistant clinical sample of *M. tuberculosis* was determined using a broth microdilution method in Middlebrook 7H9 medium supplemented with OADC, with a final inoculum of 5 × 10^2^ cells/mL. The compounds were dissolved in DMF (1.25 mg/mL) and used as a stock solution. Concentrations ranging from 1 to 1000 µg/mL were used to assess the effectiveness of the compounds. Microtiter tubes were incubated at 37 °C for 72 h, and the growth inhibition was recorded for 14 and 21 days respectively. The MIC value represents the lowest dilution of the compound at which no bacterial growth was detected.

### Culture inoculation

Each tubes were inoculated with 0.01 mL of bacterial suspension (0.5 Mc Farland standard). Medium without antimicrobial agents was inoculated with the same suspension and with a 100-fold diluted suspension, as a growing control. The tubes were sealed, and incubated at 37 °C for 28 days in a moisturized incubator. The tubes were rechecked for growth of non tuberculosis matter (NTM) for 3rd and 5th day respectively. The presence of turbidity/growth checked for 7th, 14th and 21st days respectively. The MIC values were recorded on days 14 and 21. Slides were prepared from each well for acid-fast staining. No organisms other than acid-fast bacilli were observed.

### Determination of minimum inhibitory concentration (MIC)

The evaluation of in vitro anti-mycobacterial activity of the compounds was performed against *M. tuberculosis* H_37_Rv and multidrug resistant strain (MDR).The dilutions was carried out in the broth media, i.e., Middle brooke 7H9 medium (Himedia, Mumbai) supplemented with OADC (Himedia, Mumbai) was used to determine the minimum inhibitory concentration (MIC) method. The compounds were dissolved as tabulated in Table 1. The inoculum was prepared by transferring colonies from culture to sterile water. The cell density was adjusted to 1 McFarland standard (10^8^ cells/mL). Final inoculum was made by 1:1000 dilution of the suspension with sterile water. Isoniazid was used as the drug control for the compounds. The controls used are drug free media for sterility check and the media inoculated with *M. tuberculosis H*_*37*_ *Rv* and multidrug resistant strain (MDR) for the growth patterns in drug inoculated tubes. The tubes were incubated at 37 °C. The determination of results was performed visually after 3 days of static incubation at 37 °C, after 7 days static incubation at 37 °C, and 21 days of static incubation at 37 °C. The MICs were defined as the lowest concentration of the compound at which no visible bacterial growth was observed.

**Table 1:** Synthesis of target compounds (**5a**–**5n**)

Entry	Target compounds	Method-1 (diamine + cinnamic acid)			Method-2 (2-MeBz + aldehydes)			m.p (°C)
		Time (h)	Temp (°C)	Yield (%)	Time (h)	Temp (°C)	Yield (%)	
**5a**		5	160	74	6	160	75	202–204
**5b**		5	160	68	6	160	70	178–180
**5c**		5	160	80	6	160	76	130–132
**5d**		5	150	73	6	160	76	210–212
**5e**		5	150	78	6	160	76	198–200
**5f**		5	160	74	5	160	76	>240
**5g**		5	160	68	5	150	66	100–102
**5h**		5	150	78	5	150	74	108–110
**5i**		5	160	75	5	150	75	>220
**5j**		5	150	76	6	160	74	200–220
**5k**		6	160	75	5	150	75	190–210
**5l**		5	150	75	6	160	74	202–204
**5m**		5	160	74	5	150	75	205–210
**5n**		6	150	75	6	160	75	210–220

### Molecular docking

Molecular docking studies were performed to explain the binding mode of proteins and synthesized complexes. All the compounds were (which are showing TB activity, i.e., **5d**, **5e**, **5f**, **5g**, **5i**, **5l** docked by using Autodock 4.2 software [26–29]. All the molecules were docked individually in Autodock 4.2. The three dimensional “*Crystal structure of pantothenate synthetase in complex with 2-(2-(benzofuran-2-ylsulfonylcarbamoyl)-5-methoxy-1H-indol-1-yl)acetic acid*” protein was imported to Autodock 4.2 and structurally optimized by adding hydrogens to protein allocated with kollaman charges [30–32]. After adding the hydrogens the model was saved in PDBQT format, later ligands were prepared by optimizing the torsion angles and saved them in PDBQT format. Potential binding site for the 3IVX was identified using PDBSUM. A grid was generated around to identify xyz coordinates (X = 15.137, Y = 17.850 and Z = −3.537), around binding site of 3IVX protein. Lamarckian genetic algorithm (LGA) was selected for freezing, docking and default parameters used in autodock 4.2.

## Results and discussion

### Anti-tuberculosis activity

The MIC’s were determined at different concentrations for the anti-mycobacterial activity of different compounds. The readings were taken at different intervals to see the static growth of the compounds activity against mycobacteria that is inhibiting the growth. All the compounds were tested at different dilutions the readings were taken from lowest concentration to the highest concentration in which there is no growth in any of the tubes. Thus, the compounds as the capability to inhibit the mycobacteria at different concentrations. The un known compound activity was determined with the drug control, i.e., isoniazid in which the growth in the negative control H37Rv showed no growth in these tubes whereas in MDR inoculated tube showed the visible growth pattern as tabulated in Table 2.

**Table 2:** Minimum inhibitory concentration (MIC) values of compounds **5a**–**5n**

Compound	Tube no’s	MIC dilutions for H_37_RV and MDR strain
All the 14 compounds were dissolved in DMSO	1	1000 µg/mL/1 mg/1 mL
	2	500 µg/mL
	3	250 µg/mL
	4	125 µg/mL
	5	62 µg/mL
	6	31 µg/mL
	7	15 µg/mL
	8	8 µg/mL
	9	4 µg/mL
	10	Control (without test sample)

The MIC values of selected (four) compounds BTDHA-NI, BTDHA-CU, BT-DHA, BDHA-HG, were determined for *M. tuberculosis* H_37_Rv and drug resistant clinical sample of *M. tuberculosis* using broth microdilution method in Middlebrook 7H9 medium supplemented with OADC. The compounds were tested at the concentrations of 1000, 500, 300, 250, 125, 62, 31, 15, 8, 4, µg/mL. All the compounds were showed more than 90% growth inhibition at the concentration at MIC range 4–8 µg/mL for 14 and 21 days respectively. The MIC values for four compounds are listed in Table 3.

**Table 3:** Antimycobacterial activity data of target compounds (**5a**–**5n**) by microdilution method

Compounds	MIC (lMC) MTB H_37_Rv Clinical isolate of MTB			
	15th day	21 days	15th day	21 days
**5a**	NA	NA	NA	NA
**5b**	NA	NA	NA	NA
**5c**	NA	NA	NA	NA
**5d**	8	8	8	8
**5e**	8	8	16	16
**5f**	16	16	16	16
**5g**	31	31	NA	NA
**5h**	NA	NA	NA	NA
**5i**	8	8	8	8
**5j**	NA	NA	NA	NA
**5k**	NA	NA	NA	NA
**5l**	16	16	NA	NA
**5m**	NA	NA	NA	NA
**5n**	NA	NA	NA	NA
**INH**	0.28	0.28	8	8
**R**	0.09	0.09	2	2
**S**	2	2	2	2
**E**	4	4	4	4

Controles used were first line drugs INH-isoniazid

*S* Streptomycin, *E* Ethambutol, *R* Rifampicin, *NA* compound was not active TB isolates

The screening of the synthesized compounds **5a**–**5n** against TB revealed that some of the compounds exhibited moderate to good inhibitory activity as it was evident from their MIC values, the results are listed in Table 3. Compounds **5d**, **5e**, **5f**, **5g**, **5i** and **5l** show good activity due to the substitution chloro in the 4-position of benzyl group (**5c**), furon (**5d**), thiophene (**5e**) and 2-nitro (**5i**) the most active inhibitor of tuberculosis **5d** showed MIC value of 8 μg/mL. The rest of these synthesized compounds displayed only weak activity. The remaining active compounds can be given an order for their potential activity as **5f** > **5l**. The pharmacological data obtained here may be useful for the design of novel anti-TB drugs with the skeleton of 2-heterostyrylbenzimidazole. Further studies to improve anti-TB activity and *invivo* bioassay of this class of compounds are in progress.

### Molecular modelling

**Fig. 2:**
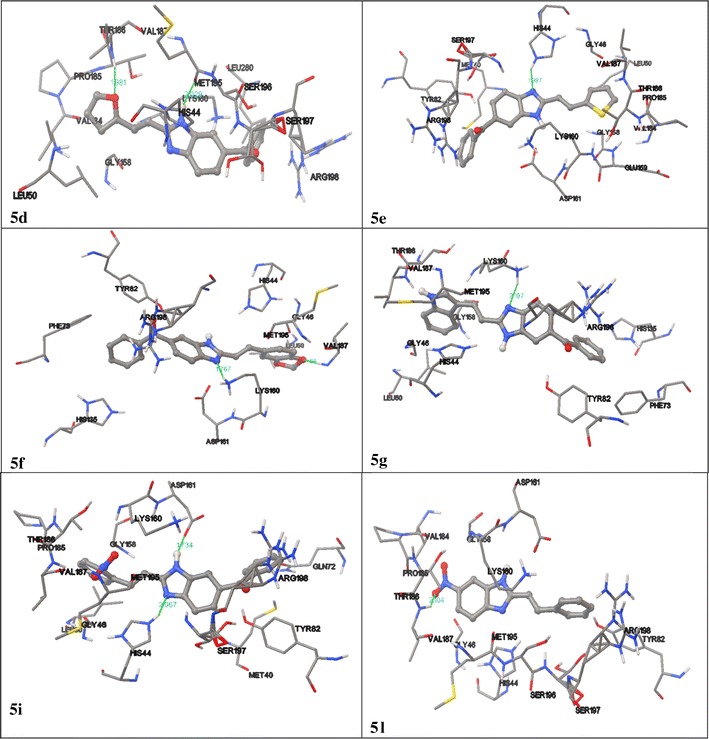
Docked confirmations of target compounds with 3IVX

Molecular docking studies of all the synthesized compounds into the binding site of a receptor and estimating the binding affinity of the ligand is a most important part of the structure based drug design process. The molecular docking results indicate that all of the studied synthesized compounds occupy an almost similar space in the binding site. Compounds (**5d**, **5e**, **5f**, **5g**, **5i**, **5l**) shows best possible binding mode against 3IVX protein is illustrated in Fig. 2 and Table 4. During the molecular docking procedure, the program selects only best fit active site pocket of the protein with respect to the ligands in order to dock them. Autodock 4.2 provides information on the binding orientation of ligands at the active site region. The docking program place both ligand and protein in different orientations, conformational positions and the lowest energy confirmations which are energetically favourable are evaluated and analyzed for interactions. Free energies of binding (ΔGb) and dissociation constants (KI) as calculated by autodock are summarized.

**Table 4:** Interactions of compounds (**5d, 5e, 5f, 5g, 5i, 5l**) with crystal structure of pantothenate synthetase in complex with (PDB id: 3IVX)

C. no	Interacting amino acids	Grid X–Y–Z coordinates	Binding energy ΔG (Kcal/Mol)	Dissociation constant (kI)
**5d**	Val287, Met195	15.137, 17.850, −3.573	−9.09	216.48
**5e**	His44	15.137, 17.850, −3.573	−8.67	441.98
**5f**	Lys160, Val187	15.137, 17.850, −3.573	−9.80	65.64
**5g**	Lys160	15.137, 17.850, −3.573	−9.67	81.08
**5i**	His44, Asp141	15.137, 17.850, −3.573	−9.59	94.13
**5l**	Val187	15.137, 17.850, −3.573	−8.53	556.07

The compound **5f** shows highest binding energy with two amino acid interactions Lys160, Val187. Figure 2 illustrates some of the synthesized compounds docked poses. Compound **5g** exhibits binding energy shows −9.67 and KI 81.08 interactions with Lys160. Almost all the target compounds show good binding energy, Π–Π interactions, Vanderwall interactions, etc. (Fig. 2). The hydrogen bonding distance of all the molecules with proteins is less than 2.0 Å. The docking score and interactions of final compounds are against the 3IVX protein illustrated in Table 4 and Fig. 2 and the order of the docking score is **5f** > **5g** > **5i** > **5l** > **5d** > **5e**, etc.

## Conclusions

Based on our bioinformatics analysis and anti mycobacterial activity of in-depth biological rationale, some of our novel anti-TB targets have been proposed as potential opportunities to improve present therapeutic treatments for this disease. The synthesis and characterization of new series of 2-heterostyrylbenzimidazole derivatives (**5a**–**5n**). Invitro* Mycobacterium tuberculosis* H_37_Rv by microdilution method using Lowenstein Jensen medium (LJ) showed that **5d**, **5e**, **5f**, **5g**, **5i**, and **5l** were slightly more active than streptomycin, ethambutol. Whereas, compound **5f** displayed the greatest activity, with a MIC value 16 μg/mL. The substituent in the aromatic ring has an important role in the biological activity and generally compounds having electron-releasing groups, such as chloro, oxygen are active. Further investigation, the designed 2-styrylbenzimidazole derivatives **5d**, **5e**, **5f**, **5g**, **5i**, and **5l** were docked and exhibited good binding energy, Π–Π interactions, Vanderwall interactions against the same receptor, the energy values are less than the standards (amoxicillin, ciprofloxacin) by employing PDB code 3IVX, and software is Autodock 4.2. So, it can be concluded that the designed compounds can be potent antitubercular agents. In future research, these 1H-benzimidazole derivatives will be synthesized and screen for their invitro anti‐tuberculosis activity.
